# Gender gaps in research productivity and recognition among elite scientists in the U.S., Canada, and South Africa

**DOI:** 10.1371/journal.pone.0240903

**Published:** 2020-10-29

**Authors:** Creso Sá, Summer Cowley, Magdalena Martinez, Nadiia Kachynska, Emma Sabzalieva

**Affiliations:** Department of Leadership, Higher, and Adult Education, Ontario Institute for Studies in Education, University of Toronto, Toronto, Ontario, Canada; Universita degli Studi di Verona, ITALY

## Abstract

This study builds upon the literature documenting gender disparities in science by investigating research productivity and recognition among elite scientists in three countries. This analysis departs from both the general comparison of researchers across organizational settings and academic appointments on one hand, and the definition of “elite” by the research outcome variables on the other, which are common in previous studies. Instead, this paper’s approach considers the stratification of scientific careers by carefully constructing matched samples of men and women holding research chairs in Canada, the United States and South Africa, along with a control group of departmental peers. The analysis is based on a unique, hand-curated dataset including 943 researchers, which allows for a systematic comparison of successful scientists vetted through similar selection mechanisms. Our results show that even among elite scientists a pattern of stratified productivity and recognition by gender remains, with more prominent gaps in recognition. Our results point to the need for gender equity initiatives in science policy to critically examine assessment criteria and evaluation mechanisms to emphasize multiple expressions of research excellence.

## Introduction

There is growing recognition in science policy debates of the interplay between gender, research productivity, and recognition in academic science [1, 2]. Gender gaps are well documented in the participation of women in the scientific workforce, in their progression through senior and leadership positions, in earning grants and awards, in publication and citation rates, and in the length of their research careers [3–7]. A recent meta-analysis shows persistent gaps in research productivity and impact between man and women, and some evidence of gender bias in the assessment of research records [8]. Studies show that women in science are under-cited [4, 9, 10], under-paid [9], under-promoted [3] and professionally under-recognized [6, 11] relative to their male counterparts. Moreover, relatively few women reach senior positions despite the growing number of women moving into doctoral studies and academic careers [3, 7, 12]. Gender inequalities continue to persist despite a number of policy initiatives and instruments at national levels aimed at redressing them [12–14].

In light of these disparities, scholars have questioned the meritocratic assumptions undergirding policy initiatives aimed at promoting research excellence [15–19]. Some highlight the role of explicit and implicit biases in the assessment of otherwise similar careers, indicating that women tend to get less recognition than men for similar research records [20, 21]. Others contend that the metrics used to gauge research performance are unfair towards women [22–24]. Awareness of these issues has prompted science policy initiatives in multiple countries. In Canada, the low representation of women among government-funded research chair holders has motivated reviews and policy measures to address gender equity [2, 25]. The promotion of gender initiatives in science has been a vital part of the European Union’s research policy for the last two decades, with a number of special projects funded by the EU to address the gender gap in science at different levels [1]. Despite many policy intentions and initiatives, the unequal recognition of scientific performance among male and female scientists is still prevalent in science [26].

Speaking to this problem, a few studies find gender gaps among the most productive scientists in their fields [27]—gaps which in some cases can be higher than for the general population of researchers [28, 29]. Some studies showing that men are disproportionally represented among the most prolific researchers in STEM disciplines suggest that women may have to accumulate more knowledge, resources, and social capital to overcome biases and achieve similar publication rates as their male counterparts [29]. These studies tend to define “star scientists” by high publication and citation rates, invariably finding men to be overrepresented in this rarified segment of the population of researchers. Hence, by definition, elite scientists in these studies are those identified by the outcome variables used to measure productivity and recognition.

This study extends these efforts by considering the meaningful role of career stratification in academia. Prevalent approaches in the literature on gender and research productivity have in some ways ignored important markers of stratification in scientific careers. Generally, studies investigate large samples of researchers by drawing on bibliometric datasets and comparing all authors with publication records (see [8] for a comprehensive meta-analysis). These studies largely ignore the material and symbolic resources researchers draw from in their research careers, which accrue from being affiliated to high-status institutional settings and holding prestigious academic appointments [30–32]. As these appointments are less accessible to women on average, these need to be accounted for in explanations of gender gaps among scientists who are considered “elite”.

To consider the stratification of academic careers, this study departs from the usual approach of using bibliometric databases to define the sample of researchers to be investigated and identifying elites by high publication and citation rates. Instead, we chose a type of academic distinction that could be used to identify elite scientists as judged by peers across national settings: research chairs. Previous studies identified productivity gains among scientists selected and funded as chairs in comparison to non-chairs [33, 34]. Sampling research chair holders allows us to isolate men and women who have been recognized as productive and meritorious scientists. As part of the general gender gap in science concerns the lower rate at which women achieve senior research positions, studying chairs minimizes that source of difference between men and women and allow us to investigate potential disparities in productivity and recognition among elite scientists.

In this paper we explore how the advantages of holding research chairs intersect with gender–do differences in productivity and recognition between men and women that have been described in previous studies hold among elite scientists who enjoy the resources and status of chairholders? To form our sample, we selected government-funded research chair programs in operation for at least five years that aim to recruit and retain senior scientists. We identified suitable programs in Canada, South Africa and in the US states of Georgia, Florida, and Kentucky. Subsequently, we created a unique, hand-curated dataset including 237 chairs and a control group of 706 non-chair peers identified from the same academic departments.

As the length of research careers is an important confounding variable in research productivity and recognition [7], our study focuses on research output over the five-year period following the appointment of research chairs. With this approach we sought to determine whether gender remains a factor in productivity and recognition during periods in the academic careers of elite scientists when they are expected, as a function of their appointments as research chairs, to be at their peak. Furthermore, we use the control group of peers to verify whether any similarities or differences between genders are unique to elite scientists. Our results show a persistent pattern of stratified productivity and recognition, which is consistent with the literature and yet intriguing considering the expected effects of prestigious academic appointments and resources on scientific careers.

## Conceptual framework

Our study is grounded in the sociology of science that has examined the relationships between social structures and research activity. One of the central contributions of sociologists of science is the investigation of how the reward system in science determines research productivity. In the idealized ‘Mertonian’ world of scientific research [35, 36], scientists are motivated by being the first to communicate an advance in knowledge and by getting the recognition awarded by the scientific community in the form of publications, citations and prizes. Peer recognition is the basic form of social reward in science from which other extrinsic rewards may be consequential, such as salary increases, career promotion and research funds, all of which usually progress in accordance with the degree of recognition achieved [37]. Thus, sociologists argue that the recognition and validation of researchers’ contributions to their field are crucial determinants of research productivity [36, 38, 39]. We frame our focus on research chairs as elite scientist through the concept of cumulative advantage.

### Cumulative advantage and stratification in science

The phenomenon when more productive scientists get more recognition that supports their further productivity has been introduced by Merton as the principle of cumulative advantage [40]. His discussion of the ‘Matthew effect’ explains how the stratification in science unfolds when, for a variety of reasons, researchers tend to choose their readings on the basis of an author’s reputation and, as a result, two publications of equal merit will be unequally recognised. Overtime, the growing prevalence of ‘Big Science’ has had an impact on the dynamics of recognition in many disciplines. Scientists connected to large scale research consortia tend to reap the benefits of higher citation rates, although authorship contributions become increasingly difficult to assess [41, 42]. Nonetheless, the principle of cumulative advantage has been a dominant theme in the studies of stratification in science. The widespread acceptance of the cumulative advantage hypothesis has been explained by its applicability in examining inequality of productivity and recognition in science [43–45].

The ‘Matthew effect’ has been confirmed through numerous empirical studies on scientific careers [46–49]. A recent study analyzed why scientists with similar backgrounds and abilities often end up achieving very different degrees of success, using data from a large academic funding program [50]. The results show that “winners just above the funding threshold accumulate more than twice as much funding during the subsequent eight years as nonwinners with near-identical review scores that fall just below the threshold” [50].

### The gender gap in scientific productivity and recognition

As noted in the introduction, the literature has long documented the ‘productivity puzzle’ [51] whereby women publish less and are less cited than men [44, 52–54]. The lower recognition and misattribution of work by female scientists, called the “Matilda Effect”, a phenomenon documented throughout history [55]. A recent meta-analysis suggests that the research productivity gap has remained consistent over generations since the mid-twentieth century [8]. Others have recently found that the growing participation of women in science over the past 60 years was accompanied by an increase of gender differences in research performance [7]. By reconstructing the publication history of over 1.5 million authors from 83 countries and 13 disciplines whose publishing career ended between 1955 and 2010, the study found that 35% of all active authors in 2005 were women comparing to only 12% of those in 1955. At the same time, the gender gap in total productivity rose from nearly 10% in the 1950s to around 35% gap in the 2000s [7]. Research also shows that the scientific awards won by women tend to be lower status [6].

The persistent evidence that men publish more than women throughout their careers has stimulated research looking for possible explanations. Thus, sociological research on academia suggests various factors which may explain gendered productivity and recognition: differences in family responsibilities [56, 57]; different time use patterns as women dedicate more time to serve on committees, teaching and mentoring students [58–60]; unequal resource allocation [61]; different patterns in academic collaboration and networking [11, 48, 62]; and gender bias in peer-review [63]. The literature documents various forms of gender stratification in academic careers [6]. Previous descriptions of changes in the representation of women in science over time point to their increased presence at lower-ranking positions, holding less-prestigious awards, and working in marginalized subdisciplines that receive less funding and lower recognition [3, 4, 6]. So, despite an increase of women in the “pipeline” of scientific disciplines, stratification manifests in the niches and career levels they reach [5]. These social differences reflect the unequal accumulation of advantage among men and women in academia, which help explain gender differences in scientific careers [51].

## Methods

This study frames research chairs as a source of advantage, as it provides material and symbolic resources to their holders who are already recognized and productive researchers. As such, they reinforce their reputation and support further scientific achievement. A focus on research chairs allows us to identify scientists of both genders who have undergone peer selection processes that designate them as part of an academic elite. These processes are arguably qualitatively and expert-based, as research chairs are usually vetted by search committees and their appointment is regulated by norms emphasizing research excellence.

Thus, we sought to identify research chair programs in different contexts to establish a sampling frame of chairholders. We focused on programs aimed at recruiting mid- to senior-level researchers in the sciences for long-term or permanent positions at the host university, employing “excellence”-related criteria. We selected two national policy initiatives—the Canada Research Chairs programs and the South Africa Research Chairs Initiative, and three state-level programs-the Georgia Research Alliance, the Kentucky Endowment Match program, and the endowment match program in the state of Florida.

The Canadian Research Chair (CRC) program was introduced in 2000 to attract and retain two thousand researchers with approximately $900 million investment from the federal government [34, 64]. As of June 2019, 1,836 CRCs have been awarded to researchers at 70 universities and affiliated institutes and hospitals (www.chairs.gc.ca). Women represent approximately 34% of chairholders in 2019. Similarly, the South African Research Chairs Initiative (SARChI) was established by the Department of Science and Technology of South Africa in 2006 to attract and retain researchers in public universities to support excellence in research [33, 65]. The program was designed based on the CRC program experience [33]. Since its implementation, the initiative has awarded 150 chairs in 21 public universities.

The three US state programs selected were part of a wave of “eminent scholars” programs introduced in the United States since the early 1990s to fund research-oriented professorships [66]. With the aim of attracting leader scholars to individual states, American research chair programs began in Georgia and spread across the United States through the 1990s and early 2000s, usually emphasizing fields of science and technology with economic potential [67].

### Dataset

Our unique dataset includes 943 researchers (237 chairs and 706 non-chair peers) along with data about their scholarly output during a five-year period. We included chairs appointed since 2000 (when the CRC program was created) in science and engineering disciplines. Through this approach we drew a sample of 237 research chairs: 264 in the US, 497 in Canada, and 182 in South Africa. Employing a matched-peers research design, we then identified a control group of 3 non-chairholders drawn from the same department as each chair, at the same academic rank, same gender (where possible) and with similar seniority (as determined by time since obtaining PhD). We gathered data from the open access self-reported resources as personal pages/CVs at university websites, LinkedIn profiles, and personal websites. The research team met during data collection to monitor the construction of the dataset and ensure consistency in the application of the selection criteria.

To measure scientific productivity, we retrieved the total number of papers published per year over a 5 year-period from Thomson Reuters Web of Science, starting one year after their appointment as chair, to capture publications more likely to have resulted from research conducted as chairholders. As explained above, we would expect to see high levels of productivity during this period as it arguably represents a high point in the chairs careers; not only have they been recognized as productive and holding potential for continued productivity and impact in their fields, but they also count on the material and symbolic resources associated with their chairs. Data from peers in each chairs’ department were gathered from the same time period to allow for direct comparison of chairs and their peers. We measure recognition through the total number of citations for the publications in this period, also retrieved from the Web of Science.

### Data analysis

To analyze the data, we performed Poisson regressions to determine the relationship between the independent variables of status as chair, discipline, country, and gender and the dependent variables of the number of articles published and the number of citations on those articles. For ease of interpretation, we calculated Incidence Rate Ratios for each relationship of interest (Table 1).

**Table 1:** Incidence rate ratios.

	log	IRR
**Articles published**		
Chairs vs. peers	.5923197***	1.808178***
Men vs. women	.1462261***	1.157458***
Male chairs vs. female chairs	.2610042***	1.298233***
Male peers vs. female peers	.0386934	1.039452
**Citations**		
Chairs vs. peers	.63985***	1.896196***
Men vs. women	.5171574***	1.677253***
Male chairs vs. female chairs	.4930763***	1.637345***
Male peers vs. female peers	.4826418***	1.620349***

* p < .05

** p < .01

*** p < .001.

In order to verify whether differing self-citation rates between male and female authors [8, 68] affect the citation counts recorded in our dataset, we conducted an additional analysis of articles from a sub-sample of the researchers in our dataset. We randomly selected 20% of all authors (n = 188) and compiled all their publications in the corresponding five-year period described above using the Web of Science search function (N = 3918). To subject these authors to a stringent test of self-citation practices by gender, we focused on papers with 1 or 2 authors, which a large-scale study of 1.5 million publications has found to tend to have the most self-citations [68]. We then categorized these papers by gender, including female solo and duo authorship (N = 34), male solo and duo authorship (N = 212), and mixed-gender authorship (N = 116). Next, we ran two simple regressions of weighted average self-citation counts on three categories of authorship gender, including male and female self-citation in the case of mixed-gender authorship.

### Variables

The outcome variables are productivity (*articles*) and recognition (*citations*). The sample had a mean number of publications of 27.43 and a standard deviation of 29.44. Mean number of citations in the sample was 256.62, with a standard deviation of 542.38. The predictor variables in this study are *chair* (chairship status) and *male* (gender), and the control variables are *discid* (discipline), *countryid* (country), and *yeardeg* (year of degree). The dummy variable *chair* describes whether a researcher is a chair or a peer and has values “1” as chair and “0” as peer. The dummy variable *male* describes the gender of the researcher and has values “1” for men and “0” for women. In the sample, there are 778 men and 165 women. The categorical variable *discid* describes the discipline of the researcher and has values “0” for life sciences, “1” for engineering and computer sciences, and “2” for physical sciences. In the sample, there are 638 researchers in the life sciences, 178 researchers in engineering and computer sciences, and 127 researchers in the physical sciences. The categorical variable *countryid* describes where the country of the researcher and has values “0” for US, “1” for Canada, and “2” for SA. The continuous variable *yeardeg* represents the year when the researcher received his or her final degree.

In our subsample of articles examing self-citation, the outcome variable is the weighted average of a self-citation dummy variable for each article (*wtselfct*). The variable *wtselfct* describes whether self-citation occurred by an author, weighted by the number of authors of that gender on that paper and can have values of “0” if no self-citation occurred, “1” if self-citation occurred once for each author, and “0.5” if self-citation occurred once for one out of two authors. The predictor variable was authorship gender category (*gencatgrp*), which had values of “0” for articles authored by one or two male authors, “1” for articles authored by one or two female authors, “2” for articles authored by a female-male group counting male self-citation in subsample regression 1 (Table 9) and counting female self-citation in subsample regression 2 (Table 2).

**Table 2:** Output 1.

Poisson regression				Number of obs = 943		
				LR chi2(7) = 3737.65		
				Prob > chi2 = 0.0000		
Log likelihood = -11402.734				Pseudo R2 = 0.1408		
articles	Coef	Std. Err.	z	P>|z|	[95% Conf. Interval]	
1.chair	.5923197	.0129326	45.8	0.000	.5669722	.6176672
1.male	.1462261	.0172582	8.47	0.000	.1124007	.1800516
discid						
1	-.0033356	.0163366	-0.20	0.838	-.0353548	.0286836
2	-.0377911	.018545	-2.04	0.042	-.0741386	-.0014436
countryid						
1	.6369511	.0166374	38.28	0.000	.6043424	.6695598
2	.3377529	.0213839	15.79	0.000	.2958412	.3796645
yeardeg	-.0006512	.000787	-0.83	0.408	-.0021937	.0008913
_cons	3.87022	1.5656	2.47	0.013	.8017008	6.938739

## Results

Before going into the main analysis, we verified whether research chairs in our sample are indeed more productive and recognized than their departmental peers. As expected, research chairs outperformed their peers comfortably. Accounting for all control variables, research chairs published 81% more articles than their non-chair peers (Tables 1 and 3) in the five-year period of interest (p<0.001), and were cited 90% more than their peers (Tables 1 and 4) in the period (p<0.001). They can thus be safely considered as elite scientists, as their productivity and recognition is well beyond those of their departmental colleagues of similar seniority and academic rank.

**Table 3:** Output 2.

Poisson regression				Number of obs = 943		
				LR chi2(7) = 42229.60		
				Prob > chi2 = 0.0000		
Log likelihood = -206597.28				Pseudo R2 = 0.0927		
articles	Coef.	Std. Err.	z	P>|z|	[95% Conf. Interval]	
1.chair	.63985	.004212	151.91	0.000	.6315946	.6481054
1.male	.5171574	.0062903	82.22	0.000	.5048286	.5294861
discid						
1	-.4537693	.0060258	-75.30	0.000	-.4655797	-.4419589
2	-.1678299	.0061404	-27.33	0.000	-.1798648	-.155795
countryid						
1	.4110643	.0050392	81.57	0.000	.4011877	.4209408
2	.0071869	.0069592	1.03	0.302	-.0064528	.0208267
yeardeg	.0013677	.0002533	5.40	0.000	.0008712	.0018642
_cons	2.042871	.5040758	4.05	0.000	1.054901	3.030841

**Table 4:** Output 3.

Poisson regression				Number of obs = 237		
				LR chi2(7) = 727.89		
				Prob > chi2 = 0.0000		
Log likelihood = -3514.5847				Pseudo R2 = 0.0938		
articles	Coef.	Std. Err.	z	P>|z|	[95% Conf. Interval]	
1.male	.2610042	.0275592	9.47	0.000	.2069891	.3150192
discid						
1	.0908272	.0263816	3.44	0.001	.0391202	.1425342
2	.1944639	.0278842	6.97	0.000	.1398119	.249116
countryid						
1	.4872713	.0270886	17.99	0.000	.4341786	.5403641
2	.2603595	.0350321	7.43	0.000	.1916978	.3290212
yeardeg	.0139618	.001387	10.07	0.000	.0112433	.0166803
_cons	-24.63855	2.75371	-8.95	0.000	-30.03572	-19.24138

Turning now to gender, we identify a pattern of stratified productivity and recognition involving male and female chairs and peers, with gendered differences in productivity and recognition differing between peers and chairs. Consistent with the literature, when considering both chairs and peers together, men in our sample generally produced more articles and were cited more times than women. All other things being equal, men published 16% more articles than women (p<0.001) (Tables 1 and 3). Furthermore, men were cited 68% more than women (p<0.001) (Tables 1 and 4).

So, do things look different among elite scientists? Among the chairs, men published 30% more articles and were cited 64% more than women (p<0.001) (Tables 1, 5 and 6). However, the difference in publication activity between men and women in the peer group was not statistically significant (p = 0.081) (Tables 1 and 7), but the former were cited 62% more than the latter (p<0.001) (Tables 1 and 8). This finding suggests that, while there is no productivity gap between male and female peers, male scientists are nonetheless more frequently cited than their female peers. However, differences in productivity are marked between genders among elite scientists, and the recognition gap remains notable.

**Table 5:** Output 4.

Poisson regression				Number of obs = 237		
				LR chi2(7) = 6707.94		
				Prob > chi2 = 0.0000		
Log likelihood = -54110.529				Pseudo R2 = 0.0584		
articles	Coef.	Std. Err.	z	P>|z|	[95% Conf. Interval]	
1.male	.4930763	.0097093	50.78	0.000	.4740463	.5121062
discid						
1	-.1446083	.009098	-15.89	0.000	-.1624401	-.1267765
2	.2519079	.0089733	28.07	0.000	.2343205	.2694952
countryid						
1	-.0197117	.0077695	-2.54	0.011	-.0349397	-.0044837
2	-.3527644	.0110466	-31.93	0.000	-.3744154	-.3311134
yeardeg	.0170197	.000437	38.95	0.000	.0161632	.0178762
_cons	-28.22374	.8678239	-32.52	0.000	-29.92464	-26.52284

**Table 6:** Output 5.

Poisson regression				Number of obs = 706		
				LR chi2(7) = 1484.25		
				Prob > chi2 = 0.0000		
Log likelihood = -7671.1288				Pseudo R2 = 0.0882		
articles	Coef.	Std. Err.	z	P>|z|	[95% Conf. Interval]	
1.male	.0386934	.0221662	1.75	0.081	-.0047516	.0821384
discid						
1	-.0528417	.020838	-2.54	0.011	-.0936834	-.0119999
2	-.1954824	.0251267	-778	0.000	-.2447298	-.1462349
countryid						
1	.7058535	.0212108	33.28	0.000	.6642811	.7474259
2	.3255295	.0274387	11.86	0.000	.2717507	.3793083
yeardeg	-.00845	.0009701	-8.71	0.000	-.0103513	-.0065486
_cons	19.46308	1.931067	10.08	0.000	15.67826	23.2479

**Table 7:** Output 6.

Poisson regression				Number of obs = 706		
				LR chi2(7) = 25059.27		
				Prob > chi2 = 0.0000		
Log likelihood = -147562.09				Pseudo R2 = 0.0783		
articles	Coef.	Std. Err.	z	P>|z|	[95% Conf. Interval]	
1.male	.4826418	.0082789	58.30	0.000	.4664155	.4988681
discid						
1	-.6577067	.0081169	-81.03	0.000	-.6736155	-.6417979
2	-.4681665	.0086166	-54.33	0.000	-.4850546	-.4512783
countryid						
1	.6704021	.0067895	98.74	0.000	.6570949	.6837092
2	.1804392	.0091664	19.68	0.000	.1624733	.1984051
yeardeg	-.0050237	.0003177	-15.81	0.000	-.0056464	-.004401
_cons	14.65639	.632671	23.17	0.000	13.4163	15.8964

**Table 8:** Article sub-sample output 1.

e	SS	df	MS	Number of obs		= 362
				F(2, 359)		= 1.43
Model	. 403690339	2	201845169	Prob > F		= 0.2399
Residual	50.5659229	359	.140852153	R-squared		= 0.0079
				Adj R-squared		= 0.0024
Total	50.9696133	361	.141190064	Root MSE		= .3753
wtselfct	Coef.	Std. Err.	t	P>|t|	[95% Conf. Interval]	
gencatgrp						
1	-.0147059	.0693333	-0.21	0.832	-.1510563	.1216446
2	.0689655	.0433433	1.59	0.112	-.0162731	.1542041
_cons	.75	.0257759	29.10	0.000	.6993093	.8006907

Finally, our testing for self-citation patterns in a sub-sample of articles showed that differences between groups (with male solo and duo authored papers as the references category) were not statistically significant (Table 9). We do not claim these represent definitive proof, as it was not the purpose of this study to investigate citation practices of research chairs; that would entail an entire study on its own. But this analysis suggests that self-citation does not seem to represent a major threat to our model.

**Table 9:** Article sub-sample output 2.

Source	SS	df	MS	Number of obs		= 362
				F(2, 160)		= 0.08
Model	.024570506	2	.012285253	Prob > F		= 0.9244
Residual	56.0831643	359	.156220513	R-squared		= 0.0004
				Adj R-squared		= -0.0051
Total	56.1077348	361	.155423088	Root MSE		= .39525
wtselfct	Coef.	Std. Err.	t	P>|t|	[95% Conf. Interval]	
gencatgrp						
1	-.0147059	.0730179	-0.20	0.840	-.1583024	.1288906
2	-.0172414	.0456467	-0.38	0.706	-.1070098	.0725271
_cons	.75	.0271457	27.63	0.000	.6966154	.8033846

## Discussion

Our results show a pattern of stratified productivity and recognition among elite scientists: men outperform women in the number of publications, and receive substantially more citations. While this pattern is consistent with general findings in the literature, our study provides important qualifications to previous studies, and points to implications for gender equity initiatives in science policy.

First, our research design employed a strategy that defines elite scientists not by the outcome variable of interest as in other studies [28, 29], but by their recognized standing in their fields as evidenced by their appointments. Their superior research performance relative to departmental peers confirms that their status is justified. Hence, our gender comparison entails an objective selection of scientists that belong in a research elite within their national, institutional, and disciplinary contexts. Among those scientists who have been regarded as sufficiently successful and productive to deserve an appointment as research chair, the gender gap remains. Therefore, our results adds to previous studies that identify an overrepresentation of men among the most productive and cited scientists [23, 28, 29, 69, 70].

Second, research shows that one explanatory factor for different productivity and citation rates is the lengh of research careers. A study examining 1.5 million researchers from 83 countries and 13 disciplines found the career length of women to be 1.7 years shorter than men’s [7]. After accounting for how long researchers actively published, annual differences in productivity became negligible (0.01 paper/year). Disparities in citation rates become about three times smaller when the researchers compared a matched sample based on career length. In contrast, our results show marked differences in productivity and recognition that cannot be attributed to the span of scientists’ careers. By looking at a delimited period of time when high productivity and recognition would be expected, as a function of research chair appointments, clear gender stratification remains.

Third, while earlier studies suggested that women's lack of recognition through citations could result from differences in publication rates [71], our study adds evidence that the gender recognition gap for elite scientists is more significant than the gender productivity gap. That is, despite sometimes relatively small differences in publication rates, men and women in this study differed greatly in the number of times their publications were cited. Among research chairs, which arguably includes well recognized scientists, the gender disparity in citation rates remain as large as that in the peer group. Considering previous studies showing that men tend to self-cite more often than women [4, 68], we analyzed the citation patterns in sub-sample of articles and did not find significant differences between genders. While admittedly limited, this analysis suggests that the large citation advantage of men likely results from other the other factors that the literature has explored that contribute to the “Matilda effect” [4, 11].

One limitation of this study is our inability to capture other indicators or research productivity, such as research funding obtained externally, PhD students supervised and graduated, and post-doctoral supervision, which go beyond the bibliometric data included in our analysis. Still, publication remains the prime currency in academic evaluation systems, providing a generally accepted measure of scientific productivity. Moreover, we are unable to explain why disparities remain among research chairs, although the literature reviewed above provides a number of possible explanations. Future research might combine the results of quantitative studies with qualitative approaches to examine in detail whether potential gender differences in how elite scientists form their research preferences, priorities, collaboration practices, and publication strategies might explain the disparities in productivity and recognition.

In conclusion, this paper adds evidence on the gendered nature of bibliographic indicators of merit among elite scientists. The use of such indicators pervades policy initiatives to promote research excellence and contribute to decisions that further reinforce gender disparities. A telling example comes from one of Canada’s federally funded programs that appointed only men to 19 highly prestigious research chairs, prompting a policy review and subsequent initiatives emphasizing gender equity [72]. Besides introducing a policy emphasis on gender representation in awarding chairs as in Canada’s case, science policy makers might critically examine the implications of relying on bibliometric indicators commonly used to establish quality or excellence when evaluating researchers. These concepts are recognized as “essentially contested” by science policy makers, and yet they often involve “inescapable simplifications” through the use of readily available quantitative indicators [73]. Acknowledging the gendered nature of bibliographic indicators of research achievement and impact allows for decision makers to thoughtfully design assessment mechanisms and evaluation criteria that emphasize multiple expressions of research excellence, going beyond the volume of publications and citations.
